# Genotype Cluster Analysis in Pathogenic* Escherichia coli* Isolates Producing Different CDT Types

**DOI:** 10.1155/2016/9237127

**Published:** 2016-03-03

**Authors:** Maryam Javadi, Mana Oloomi, Saeid Bouzari

**Affiliations:** Molecular Biology Unit, Pasteur Institute of Iran, Pasteur Avenue, Tehran 13164, Iran

## Abstract

Diarrheagenic and uropathogenic* E. coli* types are mainly characterized by the expression of distinctive bacterial virulent factors.* stx1*,* stx2* (Shiga toxins), and* cdt* (cytolethal distending toxin) genes have been acquired by horizontal gene transfer. Some virulent genes such as* espP* (serine protease),* etpD* (part of secretion pathway), and* katP* (catalase-peroxidase), or* sfpA* gene (Sfp fimbriae), are on plasmids and the others like* fliC *(flagellin) and the* fimH* gene (fimbriae type-I) are located on chromosome. Genomic pathogenicity islands (PAIs) carry some virulent genes such as* hly* gene. To determine the existence of virulence genes in* cdt* clinical isolates, genes including* stx1*,* stx2*,* cdt*,* hly*,* espP*,* katP*,* sfpA*,* etpD*,* fliC*, and* fimH* were assessed by Polymerase Chain Reaction (PCR). The most prevalent isolates for* etpD* and* katP* genes were 85.7% in* cdtII*.* katP* gene was also observed 83.3% in* cdtI*. However, in 42.85% of* cdtIII* isolates,* espP* gene was the most detected. Moreover,* hly* gene was also the most prominent gene in* cdtIII* (71.42%).* sfpA gene* was observed in 66.6% of* cdtV*.* stx1* gene was detected in 100% of* cdtII*,* cdtIV*, and* cdtV* types. Presence and pattern of virulence genes were considered among* cdt* positive isotypes and used for their clustering and profiling.

## 1. Introduction 

*Escherichia coli*  (*E. coli*) are known as a commonly colonizing bacteria of the human intestinal tract. Foreign DNA including plasmids, pathogenicity islands (PAIs), transposons, and phages are acquired through the horizontal gene transfer by an ancient nonpathogenic* E. coli* strain and led to development of specific pathotypes. However, the genomic background has a pivotal role in evolutionary pathways. Pathogenic* E. coli* strains are classified based on repertoires of virulence factors and the most common diseases associated with them.

Shiga toxins are a family of related toxins with two major groups, Stx1 and Stx2, expressed by genes considered to be horizontally acquired by bacteriophages. Shiga toxin encoded by* stx1* and* stx2* genes is an A-B type toxin that inhibits protein synthesis and causes hemorrhagic colitis and hemolytic-uremic syndrome. The* stx* genes are located in the genome of heterogeneous lytic (*stx2*) or cryptic (*stx1*) lambdoid phages [1–4]. Cytolethal distending toxins (CDTs) were the firstly recognized bacterial toxins that block the eukaryotic cell cycle, suppress cell proliferation, and eventually lead to cell death. A CDT is a tripartite holotoxin in which* cdtB* is the active subunit and has DNAase-I-like activity [5]. Five different CDTs (I–V) have been reported for* E. coli* so far, and they were designated in order of publications. CDT production has been associated with pathogenic* E. coli*. The presence of* cdt* genes in different bacterial species and the analysis of the DNA in the vicinity of the* cdt* genes suggest that the toxin has been acquired from heterogenic species by horizontal gene transfer. However, the probable phylogenetic origin (or ancestor) has still remained elusive. Interestingly, the phage and the corresponding insertion sequence remnants were found nearby the* E. coli cdt* genes. These data suggest that* cdt* genes were acquired by horizontal transfer events at some point and evolved separately since then [5, 6].

As with* stx* gene, some types of* cdt* genes are horizontally acquired by phages in* E. coli* [6, 7].

It is now evident that some virulent genes are located on a large virulent plasmid (pO157) in pathogenic* E. coli*, among which are the extracellular serine protease gene (*espP*), catalase-peroxidase gene (*katP*), and type II secretion pathway protein D (*etpD*). Besides different sizes of this plasmid were reported and may not contain all these 3 genes which indicate independency of genetic exchanges through the horizontal gene transfer process. EspP can be grouped into the autotransporter proteins family and characterized by catalytically active serine residue in the active center. EspP cleaves pepsin and human coagulation factor V [8–11]. Catalase-peroxidase gene (*katP*) encodes a protein which shows bifunctional catalase and peroxidase activity [12]. This enzyme is expressed by pathogenic strains and has been thought to protect these pathogens from oxidative damage caused by reactive oxygen molecules produced by phagocytes or other host cells during the infection process [13]. Type II secretion pathway protein D encoded by* etpD* gene is another pathogenic factor encoded on the aforementioned plasmid [14].

A cluster of six genes, termed* sfp* including* sfpA* gene, are located on another plasmid, pSFO157, in some pathogenic* E. coli* strains. This genomic cluster mediates mannose-resistant hemagglutination and expression of a novel type of fimbriae, Sfp fimbriae, which is 3–5 nm in diameter, the major subunit of which is SfpA [15, 16].

Type 1 fimbriae, encoded by a chromosomally located* fim* gene cluster, are the most common adhesive organelles of* Escherichia coli*. Fimbriae-mediated adherence, which facilitates colonization and survival in host cells, plays a significant role in pathogenesis. Type 1 fimbriae consist of a major structural subunit (FimA) and several minor components, including adhesin (FimH) [17, 18].* fliC* gene encoding the flagellin subunit is located on chromosome and could be considered as genomic background. Flagella facilitate bacterial movement and have a vital role in bacterial distribution in intestine and host tissues [19].

Pathogenicity islands (PAIs) are a subgroup of genomic islands that carry one or more virulent genes and are present in the genome of a pathogenic bacterium but absent from the genomes of nonpathogenic species. PAIs occupy relatively large genomic regions. The regions carrying hemolysin gene (*hly*) is located in PAI of* E. coli* chromosome [20–22].

In this study, the occurrence of virulent plasmid-borne genes, pathogenicity island, and chromosomally encoded genes in CDT-producing* E. coli* strains was investigated.

## 2. Materials and Methods

### 2.1. Bacterial Strains

In this study, 30 CDT-producing strains were investigated. The strains were isolated from clinical diarrheal patients [25, 26] and were cultured overnight at 37°C in Luria Bertani (LB) medium.

### 2.2. DNA Extraction

Template DNA extraction was performed by Phenol-Chloroform assay (1 mL from cultured LB centrifuged at 14000 rpm for 3 minute), and then the pellet was dissolved in 1 mL of 5 mM Tris-HCl pH = 6.8, centrifuged at 10000 rpm for 2 minutes. The pellet was then resuspended in 350 *μ*L of 5 mM Tris-HCl pH = 6.8 with 25 *μ*L lysozyme enzyme (5 mg/mL) and 25 *μ*L of 1 M Tris-HCl, pH: 8.8, and incubated for 15 minutes in room temperature. Next, 15 *μ*L 0.5 M EDTA was added with 40 *μ*L SDS10% and 10 *μ*L RNase enzyme (20 mg/mL), mixed gently, and treated at 37°C for 2-3 hours. After adding 5 *μ*L proteinase K (20 mg/mL), the samples were incubated overnight at 37°C and, afterwards, heated for 5 minutes at 55°C. Then, 400 *μ*L phenol was added and mixed gently and 400 *μ*L chloroform was added, mixed vigorously or by vortex, and centrifuged for 20 minutes (14000 rpm). The upper phase was collected, and 800 *μ*L cold ethanol 96% was added and left at room temperature for 15–30 minutes and then was centrifuged at 14000 rpm for 15 minutes. The upper phase was discarded and the pellet was washed 1-2 times, dried, and dissolved in 50 *μ*L TE (1x) and then heated at 75°C for 15 minutes.

In addition, elution was performed using TE (1x) buffer. Totally, the concentration of templates was adjusted to 100 ng/*μ*L.

### 2.3. Amplification of Target Genes and PCR Conditions

Virulence-associated genes, including* fliC*,* fimH*,* stx1*,* stx2*,* etpD*,* espP*,* sfpA*,* katP*, and* hly* genes, were assessed by Polymerase Chain Reaction (PCR).* cdt* typing based on specific primers was performed. In Table 1 the nucleotide sequence of primers used for amplification of target genes is depicted.

PCR samples were prepared in a total volume of 25 *μ*L (PCR buffer: (10x) buffer/CinnaGen Co, dNTP: 25 mM/Fermentas, Taq DNA polymerase: 5 units per *μ*L/CinnaGen Co, MgCl_2_: 50 mM/CinnaGen Co., D.D.W., Template: extracted genomic DNA (100 ng/*μ*L), Thermocycler: SENSOQUEST Labcycler).

### 2.4. Cloning and Sequencing

In this study, we cloned some positive samples and sequenced the PCR products. After PCR optimization and gene amplification, gene extraction from gel agarose was done with “Core one*™* Gel extraction kit GE-100, CoreBioSystem Co. Ltd.” We also utilized pTZ57R/T vector for ligation process which was done with InsTA clone*™* PCR Cloning Kit (Fermentas). Recombinant cells were cultured on plates containing Ampicillin (500 mg/mL).

### 2.5. Hierarchical Clustering

Virulence-associated genes of strains were compared based on dendogram illustration. A hierarchical clustering analysis was performed and dendrogram was constructed by IBM SPSS Statistics software (Version 20). All virulent genes regarded as variables and statistics setting was set on Agglomeration schedule. Cluster method was between-groups linkage by measuring squared Euclidean distance.

## 3. Results

### 3.1. Virulent Genes Detection

Existence of virulence genes, including* espP*,* etpD*,* katP*,* sfpA*,* hly*,* fliC*,* fimH*,* stx1*,* stx2*, and* cdt* genes, was assessed by PCR (Table 2).

All strains were* cdt* positive and regarding* cdt* types 6 strains were* cdt-I* (20%), 7 strains (23.3%) were* cdt-II*,* cdt-III*, and* cdt-IV*, and 3 strains were recognized as* cdt-V* (10%).

In the present study,* stx1* gene was present in 86.7% of clinical isolates. However, the* stx2* gene was detected in 13.3% of strains.* stx1* gene was also detected in 83.3% of* cdt*-type-*I* producing strains, while it was detected in all the* cdt*-type* II*,* IV*,* V* strains and in 57.1% of* cdt*-*III* isolates (Table 3).* stx2* gene was present at 33.3% of* cdt*-type *I* strains, 14.3% in* cdt-*type* III*,* IV* strains, while in CDT-*II* and CDT-*V* producing isolates it was not observed.

*hly* gene was not detected in any of CDT-I-producing strains. However, it was detected in 14.3% of* cdt*-*II* strains, 71.4% of* cdt*-*III* strains, 57.1% of* cdt*-*IV* strains, and 66.7% of CDT-V-producing isolates (Table 3). In this clinical isolates, 53.3%* etpD*, 63.3%* katP*,and 23.3%* espP *genes were detected. In the present study,* etpD* gene was observed in 33.3% of* cdt*-type I, 85.7% of* cdt*-type II, 28.6% of* cdt*-type III, 57.1% of* cdt*-type IV, and 66.7% of* cdt*-type V strains.* espP* gene was present in 16.7% of* cdt*-type I, 42.9% of CDT-III-producing, 28.6% of* cdt*-type IV, 33.3% of* cdt*-type V strains, while, in CDT-II-producing isolates, the gene was not detected. In addition,* katP* gene was detected in 83.3% of* cdt*-type I, in 85.7% of* cdt*-type II, in 57.1% of CDT-III- and CDT-IV-producing strains, while, this gene was not observed in CDT-V-producing strains (Table 3).

Occurrence of virulent genes,* espP*,* etpD*,* katP,* and* sfpA,* was classified as an A–M pattern which was based on the existence of plasmid encoding genes (Table 4).

The most prominent F profile with 20% frequency was repeated 6 times (*etpD/katP*). The absence of plasmid genes was shown in 10% of strains. Frequency of all the plasmid born associated genes with L profile was only 3.33% (*espP/etpD/katP/sfpA*).

The higher prevalence of profile F (*etpD/katP*) is considerable (20%) and then profile C (*katP*) is more prevalent (16.68%). The frequency of profiles B (*etpD*), D (*espP/katP*), G (*etpD/sfpA*), and M (absence of plasmid-borne genes) was 10%. The frequency of other remaining profiles was 3.33% exceptionally and profile I (*etpD/katP/sfpA*) is 6.67%.

In* cdt-I* group, 50% I profile (*etpD/katP/sfpA*) was shown, and in 40% of strains, C profile (*katP*) was demonstrated. In* cdt-II* group, 50% strains also showed I profile (*etpD/katP/sfpA*) and in 66.67% of strains F profile (*etpD/katP*) (the most prominent profile) was detected. The prominent profiles in* cdt-III* group were 100% E profile (*espP/sfpA*) and 66.67% D profile (*espP/katP*). H, K, and L profiles are the most frequent profiles (100%) in* cdt-IV* group. In* cdt-V* group, 100% of strains have A profile (*espP*) and 66.67% showed G profile (*etpD/sfpA*). In each* cdt* group specific plasmid born profile was shown in Table 5.

The most prominent gene in each group was determined.* katP* plasmid gene was presented 83.3% in* cdt-I* and 85.7% in* cdt-II* groups. The* etpD* gene presentation was 85.7% in* cdt-II* group and 66.66% in* cdt-IV* group;* espP* presentation was 42.85% and 33.33% in* cdt-III* and* cdt-V* group, respectively. The* sfpA* presentation in* cdt-IV* and* cd-tV* group was 42.85% and 66.66%, respectively.

### 3.2. Dendogram Clustering of Strains

**Figure 1:**
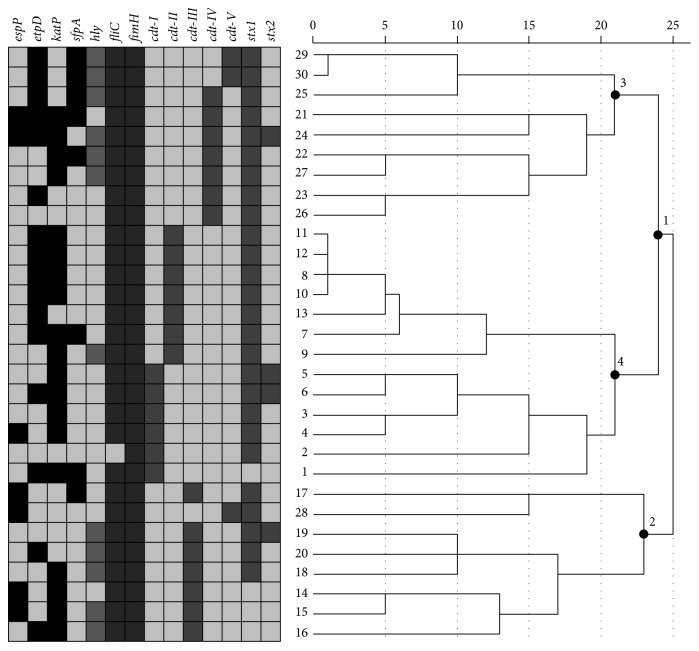
Dendrogram using average linkage between groups: This figure illustrates hierarchical clustering analyses between 30 examined clinical isolates which are clustered based on virulence-associated factors including virulent plasmid, phage, chromosomal, and pathogenicity island genes. At the end of each branch the numbers indicate strains, as moving to the right relative distance becomes more apparent. Distances are more distinct by using a 0 to 25 scale along the top of the chart. Depicted table which is in front of the tree illustrates the comparative patterns of cooccurrence and presence of virulent genes in different strains (light gray scale shows gene loss and different dark gray scales show different virulent genes genesis). Defined pattern can be observed for each* cdt*-type isolates based on other virulent genes.

In Figure 1, hierarchical clustering analysis based on average linkage and rescaled distance cluster combine was done. The pattern of virulence gene association was compared and the linkage of these strains was illustrated. In this dendogram, association of* cdt-I*,* cdt-II* (branch 4) and genetic linkage of* cdt-IV*,* cdt-V* (branch 3) groups was shown in branch 1. The genotype association of* cdt-III* was also shown in a completely separated branch (branch 2).

## 4. Discussion

In the current study, we analyzed different* E. coli* strains isolated from clinical diarrheal patients in order to obtain evidence of existence between virulence genotype and different* cdt* types. It has been shown that CDT production has been associated with pathogenic* E. coli*. Both the low prevalence of* cdt* genes and their association with other virulent genes suggest that the* cdt* genes are acquired independently in a number of* E. coli* lineages, possibly as a result of horizontal gene transfer [7]. Five different CDTs have been reported for* E. coli*, so far [5]. It has also been shown that* cdt-I* and* cdt-IV* genes appeared to belong to the same phylogenetic lineage, whereas the* cdt-II*,* cdt-III,* and* cdt-V* genes are clustered together in another lineage. This clustering profile was based on genomic extend diversity. In our study, regarding the assessed virulent genes two branches were first separated into branch 1 (including* cdt-I*,* cdt-II* and* cdt-IV*,* cdt-V* genes) and branch 2,* cdt-III* genes. These data suggest that* cdt* genes were acquired by horizontal transfer events at some point and evolved separately since then [5]. Our study shows complete separation of* cdt-III* from other cdt types that agree with the finding that this group originated from animals and the other groups are more belonging to human. Furthermore,* cdt-I*,* cdt-II* (branch 4) and* cdt-IV*,* cdt-V* (branch 3) groups are more similar regarding virulence genes and originated from same progeny.

It is now evident that* hly* gene is located on a PAI and encodes alpha-hemolysin.

The production of hemolysin in* cdt-III-*,* cdt-IV*-producing human and animal pathogenic* E. coli* strains was observed frequently [5]. Thus, not surprisingly,* hly* was detected in 40% of our clinical isolates. In concurrence with other studies, the prevalence of* hly* gene in* cdt*-type III, IV, V was more than other isolates. These results demonstrate that, possibly, there could be a relationship between the existence of* hly* gene and the type of* cdt* gene in clinical* E. coli* isolates.

*fliC* and* fim* are the two chromosomally located genes analyzed in our study.* fliC* gene was detected in 96.7% of our isolates.* fliC* gene encodes the flagellin subunit and could be considered as genomic background of* E. coli*. Thus it could be expected to detect* fliC* in the most of our isolates. Similar to our findings, all the isolates have* fliC* gene in an experiment performed in Finland in 2006 [23]. Type 1 fimbriae are also encoded by the chromosomally located* fim* gene cluster. The presence of* fim* DNA sequences is common among* E. coli* strains. In fact, the majority of clinical isolates, both virulent and avirulent, could be induced to express type 1 fimbriae.

Among pathogenic* E. coli*, the existence of a large virulent plasmid (pO157) has been observed. The* etpD*,* katP*, and* espP* genes are located on this plasmid. pO157 plasmid is mainly associated with EHEC and ETEC strains. In our study, 53.3% of clinical isolates harbor* etpD*, 63.3%* katP* and 23.3%* espP* genes. There is a relation between the occurrence of* stx* genes and these virulent plasmid-associated genes [24]. Moreover, PCR analysis revealed a close relationship between the occurrence of plasmid-borne* katP* gene and* stx* gene in pathogenic* E. coli* [12]. Most of* katP+* strains belong to shiga toxin-producing* E. coli* [24]. Our results were similar to the findings by Beutin et al., 2005 [24, 27]. Based on our findings, it could be deduced that* katP* gene is mostly present in CDT-I- and CDT-II-producing strains.

ESpP, which possesses human coagulation factor V and pepsin A proteolytic activity, is the significant marker of virulence in shiga toxin-producing strains [28]. High frequency of* espP* in CDT-III-producing isolates is considerable.

Alpha-hemolysin is frequently associated with human uropathogenic* E. coli* (UPEC); furthermore related encoding PAI is also so instable and the operon could be located on either a plasmid or the chromosome [22, 29]. Besides urinary tract infection (UTI) is caused predominantly by type 1-fimbriated UPEC and initial binding is mediated by the FimH adhesin of the mentioned fimbriae [30]. In our study in 100% of* hly* ^*+*^ strains,* fimH* gene was detected. In addition, all* hly* ^*+*^ strains possess one or more of plasmid pO157 genes including* etpD*,* katP*, and* espP.* These genes plus* stx* gene are one of the EHEC and STEC characteristics although* espP* gene is common in EPEC and EHEC [14, 31]. Simultaneous presence of these genes indicates that our isolates obtain* hly* operon and relevant PAI through the horizontal gene transfer. In addition, in evolutionary pathway, isolates by achieving the* cdt* genes improve their pathogenicity.

Our findings demonstrate that considering virulence genes CDT-producing strains belong to the heterogeneous group. The branching-type nature of dendrogram allows us to cluster the strains at various levels. Moreover, it gives an idea of how great the distance is between the cases that are clustered in a particular step, using a 0 to 25 scale along the top of the chart (Figure 1). Strains which are clustered as a particular group or close together in dendrogram are likely to have similar characteristics while possess their own unique genotype and genomic content. In the same cluster, one can also observe a relatively unique pattern of virulent genes which is shown in Figure 1. For instance, each distinct* cdt*-type group, by possessing a particular* cdt* gene as genomic backbone, has an approximately similar pattern based on other virulent genes. For example, profile F (*etpD/katP*) is more prevalent in* cdt*-type II group or* hly* gene is prevalent in* cdt*-type III isolates.

In addition, this phenomenon is also observed in each group belonging to a particular plasmid-profile, so that* cdt*-*V* gene is the most prevalent in profile G (*etpD/sfpA*). This evidence further confirms that horizontal gene transfer could occur among pathogenic strains.

Association of* cdt-I*,* cdt-II* and* cdt-IV*,* cdt-V* genotype groups is depicted in the dendogram. Accordingly,* cdt-III* genotype association has been shown in a separated branch. On the other hand, from 3 strains of* cdt-V*, two strains were associated with* cdt-IV* while one strain was associated with* cdt-III.*

These findings may indicate that CDT-producing strains may have originated from a common ancestor but during their evolution by horizontal gene transfer, and they departed from each other.

## Figures and Tables

**Table 1:** Nucleotide sequence of primers for amplification of target genes.

Target gene	Nucleotide sequence (forward/reverse)	Size (bp)	Reference
*sfpA*	5′-AGC CAA GGC CAA GGG ATT ATT A-3′ 5′-TTA GCA ACA GCA GTG AAG TCT C-3′	440-bp	[23]

*espP*	5′-AAA CAG CAG GCA CTT GAA CG-3′ 5′-GGA GTC GTC AGT CAG TAG AT-3′	1830-bp	[24]

*katP*	5′-CTT CCT GTT CTG CTG ATT CTT CTG G-3′ 5′-AAC TTA TTT CTC GCA TCA TCC-3′	2125-bp	[24]

*etpD*	5′-CGT CAG GAG GAT GTT CAG-3′ 5′-CGA CTG CAC CTG TTC CTG ATT A-3′	1062-bp	[24]

*fliC*	5′-CCA GTC ATT AAT ACA AAC AGC C-3′ 5′-GAC ATA TTA GAG ACT TCG GT-3′	1600-bp	[23]

*fimH*	5′-CTT ATG GCG GCG TGT TAT CTA-3′ 5′-GCC AGT AGG CAC TAC CAC ATC-3′	289-bp	[1]

*hly*	5′-AGG TTC TTG GGC ATG TAT CCT-3′ 5′-TTG CTT TGC AGA CTG CAG TGT-3′	555-bp	[25]

*stx1*	5′-CGA TGT TAC GGT TTG TTA CTG TGA CAG C-3′ 5′-AAT GCC ACG CTT CCC AGA ATT G-3′	244-bp	[25]

*stx2*	5′-GTT TTG ACC ATC TTC GTC TGA TTA TTG AG-3′ 5′-AGC GTA AGG CTT CTG CTG TGA C-3′	324-bp	[25]

*cdt-I*	5′-CAA TAG TCG CCC ACA GGA-3′ 5′-ATA ATC AAG AAC ACC ACC AC-3′	411-bp	[26]

*cdt-II*	5′-GAA AGT AAA TGG AAT ATA AAT GTC CG-3′ 5′-TTT GTG TTG CCG CCG CTG GTG AAA-3′	556-bp	[26]

*cdt-III*	5′-GAA AGT AAA TGG AAT ATA AAT GTC CG-3′ 5′-TTT GTG TCG GTG CAG CAG GGA AAA-3′	555-bp	[26]

*cdt-IV*	5′-CCT GAT GGT TCA GGA GGC TGG TTC-3′ 5′-TTG CTC CAG AAT CTA TAC CT-3′	350-bp	[26]

*cdt-V*	5′-AGC ACC CGC AGT ATC TTT GA-3′ 5′-AGC CTC TTT TAT CGT CTG GA-3′	1363-bp	[26]

**Table 2:** The occurrence of virulence-associated genes including plasmid, Pathogenicity Island, chromosomal and phage genes. (+) indicates positive PCR product and the existence of mentioned gene while (−) indicates negative PCR product.

Strain	* espP*	* etpD*	* katP*	* sfpA*	* Hly*	* fliC*	* fimH*	* cdt-I*	* cdt-II*	* cdt-III*	* cdt-IV*	*cdt-V*	*stx1*	* stx2*
1	−	+	+	+	−	+	+	+	−	−	−	−	−	−
2	−	−	−	−	−	−	+	+	−	−	−	−	+	−
3	−	−	+	−	−	+	+	+	−	−	−	−	+	−
4	+	−	+	−	−	+	+	+	−	−	−	−	+	−
5	−	−	+	−	−	+	+	+	−	−	−	−	+	+
6	−	+	+	−	−	+	+	+	−	−	−	−	+	+
7	−	+	+	+	−	+	+	−	+	−	−	−	+	−
8	−	+	+	−	−	+	+	−	+	−	−	−	+	−
9	−	−	+	−	+	+	+	−	+	−	−	−	+	−
10	−	+	+	−	−	+	+	−	+	−	−	−	+	−
11	−	+	+	−	−	+	+	−	+	−	−	−	+	−
12	−	+	+	−	−	+	+	−	+	−	−	−	+	−
13	−	+	−	−	−	+	+	−	+	−	−	−	+	−
14	+	−	+	−	−	+	+	−	−	+	−	−	−	−
15	+	−	+	−	+	+	+	−	−	+	−	−	−	−
16	−	+	+	−	+	+	+	−	−	+	−	−	−	−
17	+	−	−	+	−	+	+	−	−	+	−	−	+	−
18	−	−	+	−	+	+	+	−	−	+	−	−	+	−
19	−	−	−	−	+	+	+	−	−	+	−	−	+	+
20	−	+	−	−	+	+	+	−	−	+	−	−	+	−
21	+	+	+	+	−	+	+	−	−	−	+	−	+	−
22	−	−	+	+	+	+	+	−	−	−	+	−	+	−
23	−	+	−	−	−	+	+	−	−	−	+	−	+	−
24	+	+	+	−	+	+	+	−	−	−	+	−	+	+
25	−	+	−	+	+	+	+	−	−	−	+	−	+	−
26	−	−	−	−	−	+	+	−	−	−	+	−	+	−
27	−	−	+	−	+	+	+	−	−	−	+	−	+	−
28	+	−	−	−	−	+	+	−	−	−	−	+	+	−
29	−	+	−	+	+	+	+	−	−	−	−	+	+	−
30	−	+	−	+	+	+	+	−	−	−	−	+	+	−

**Table 3:** The frequency of target genes in different *cdt*-type isolates.

*cdt* type	Target gene						
	*katP*	*etpD*	*espP*	*sfpA*	*stx1*	*stx2*	*hly*
*cdt*-type *I*	83.33%	33.33%	16.6%	16.66%	83.33%	33.33%	0%
*cdt*-type *II*	85.7%	85.7%	0%	14.28%	100%	0%	14.28%
*cdt*-type *III*	57.14%	28.57%	42.85%	14.28%	57.14%	14.28%	71.42%
*cdt*-type *IV*	57.14%	57.4%	28.57%	42.85%	100%	14.28%	57.14%
*cdt*-type *V*	0%	66.66%	33.33%	66.66%	100%	0%	66.66%

**Table 4:** Different *cdt*-type strains and related genotype based on other virulent genes and profile designation based on cooccurrence of plasmid-borne genes. *espP*: profile A, *etpD*: profile B, *katP*: profile C, *espP/katP*: profileD, *espP/sfpA*: profile E, *etpD/katP*: profile F, *etpD/sfpA*: profile G, *katP/sfpA*: profile H, *etpD/katP/sfpA*: profile I, *espP/etpD/katP*: profile K, *espP/etpD/katP/sfpA*: profile L, and profile M: Indicates the absence of plasmid genes and presence of chromosomal genes (*fimH*, *fliC*, *hly*, *stx1, or stx2*).

Strain	*cdt*-type	Genotype based on virulence-related genes	Profile designation based on plasmid-borne genes
1	*cdt-I *	*fimH*,* fliC*, *etpD*,* katP*,* sfpA*	I
2	*cdt-I *	*fimH*,* stx1*	M
3	*cdt-I *	*fimH*,* fliC*,* stx1*, *katP*	C
4	*cdt-I *	*fimH*,* fliC*,* stx1*, *espP*,* katP*	D
5	*cdt-I *	*fimH*,* fliC*,* stx1*,* stx2*, *katP*	C
6	*cdt-I *	*fimH*,* fliC*,* stx1*,* stx2*, *etpD*,* katP*	F
7	*cdt-II *	*fimH*,* fliC*,* stx1*,* etpD*,* katP*,* sfpA*	I
8	*cdt-II *	*fimH*,* fliC*,* stx1*,* etpD*,* katP*	F
9	*cdt-II *	*fimH*,* fliC*,* hly*,* stx1*,* katP*	C
10	*cdt-II *	*fimH*,* fliC*,* stx1*,* etpD*,* katP*	F
11	*cdt-II *	*fimH*,* fliC*,* stx1*,* etpD*,* katP*	F
12	*cdt-II *	*fimH*,* fliC*,* stx1*,* etpD*,* katP*	F
13	*cdt-II *	*fimH*,* fliC*,* stx1*, *etpD*	B
14	*cdt-III *	*fimH*,* fliC*, *espP*,* katP*	D
15	*cdt-III *	*fimH*,* fliC*,* hly*,* espP*,* katP*	D
16	*cdt-III *	*fimH*,* fliC*,* hly*, *etpD*,* katP*	F
17	*cdt-III *	*fimH*,* fliC*,* stx1*, *espP*,* sfpA*	E
18	*cdt-III *	*fimH*,* fliC*,* hly*,* stx1*, *katP*	C
19	*cdt-III *	*fimH*,* fliC*,* hly*,* stx1*,* stx2*	M
20	*cdt-III *	*fimH*,* fliC*,* hly*,* stx1*, *etpD*	B
21	*cdt-IV *	*fimH*,* fliC*,* stx1*, *espP*,* etpD*,* katP*,* sfpA*	L
22	*cdt-IV *	*fimH*,* fliC*,* hly*,* stx1*, *katP*,* sfpA*	H
23	*cdt-IV *	*fimH*,* fliC*,* stx1*, *etpD*	B
24	*cdt-IV *	*fimH*,* fliC*,* hly*,* stx1*,* stx2*,* espP*,* etpD*,* katP*	K
25	*cdt-IV *	*fimH*,* fliC*,* hly*,* stx1*,* etpD*,* sfpA*	G
26	*cdt-IV *	*fimH*,* fliC*,* stx1*	M
27	*cdt-IV *	*fimH*,* fliC*,* hly*,* stx1*,* katP*	C
28	*cdt-V *	*fimH*,* fliC*,* stx1*,* espP*	A
29	*cdt-V *	*fimH*,* fliC*,* hly*,* stx1*, *etpD*,* sfpA*	G
30	*cdt-V *	*fimH*,* fliC*,* hly*,* stx1*, *etpD*,* sfpA*	G

**Table 5:** The frequency of different *cdt*-types based on virulent plasmid-borne genes in isolates totally.

Profile	Target gene				
	*cdt-I*	*cdt-II*	*cdt-III*	*cdt-IV*	*cdt-V*
A	0%	0%	0%	0%	100%
B	0%	33.33%	33.33%	33.33%	0%
C	40%	20%	20%	20%	0%
D	33.33%	0%	66.67%	0%	0%
E	0%	0%	100%	0%	0%
F	16.67%	66.67%	16.67%	0%	0%
G	0%	0%	0%	33.33%	66.67%
H	0%	0%	0%	100%	0%
I	50%	50%	0%	0%	0%
K	0%	0%	0%	100%	0%
L	0%	0%	0%	100%	0%
M	33.33%	0%	33.33%	33.33%	0%
